# Anti‐Metastatic Effects of Aprepitant on Glioblastoma Cells: Targeting the Substance P/Neurokinin‐1 Pathway

**DOI:** 10.1002/cam4.71148

**Published:** 2025-08-14

**Authors:** Selin Engür‐Öztürk, Elif Kaya‐Tilki, Miriş Dikmen

**Affiliations:** ^1^ Department of Pharmacy Services Tavas Vocational School of Health Services, Pamukkale University Denizli Turkey; ^2^ Department of Pharmacology Faculty of Pharmacy, Anadolu University Eskişehir Turkey; ^3^ Department of Pharmaceutical Microbiology, Faculty of Pharmacy Anadolu University Eskişehir Turkey

**Keywords:** aprepitant, NK‐1 receptor antagonist, substance P, U87‐MG

## Abstract

**Background:**

Glioblastoma (GB) is an aggressive brain tumor characterized by rapid proliferation, invasion, and resistance to therapy. The substance P (SP)/neurokinin‐1 receptor (NK‐1R) pathway contributes to GB progression by promoting angiogenesis, inflammation, and extracellular matrix remodeling. Aprepitant, an FDA‐approved NK‐1R antagonist, has shown potential as a therapeutic agent in glioma treatment.

**Methods:**

This study investigates the anti‐metastatic effects of aprepitant on U87‐MG glioblastoma cells. Cell viability, migration, and invasion were evaluated using WST‐1, real‐time cell analysis, and Oris migration assays. Gene and protein expression levels were assessed by RT‐PCR, ELISA, and microarray analysis.

**Results:**

Aprepitant significantly reduced glioma cell proliferation, migration, and invasion in a dose‐dependent manner. It downregulated pro‐tumorigenic mediators such as VEGF, NF‐kB, TNF‐α, IL‐6, IL‐8, CCL3, CXCL3, and TRIM5, indicating suppression of angiogenesis, inflammation, and chemotaxis pathways.

**Discussion:**

These findings underscore the potent anti‐metastatic and anti‐angiogenic effects of NK‐1R antagonism in glioblastoma and highlight aprepitant as a promising candidate for future in vivo and *in ovo* therapeutic investigations targeting extracellular matrix (ECM) remodeling, chemotaxis, and angiogenesis.

## 1 Introduction

Glioblastoma (GB) is one of the most aggressive tumors of the central nervous system, characterized by its highly invasive and metastatic nature. Despite advances in therapeutic strategies, including surgical resection, radiotherapy, and chemotherapy with temozolomide, the prognosis remains dismal, with a median survival of approximately 12–15 months following diagnosis [1, 2]. The resistance of GB to conventional therapies stems from its extensive heterogeneity, ability to infiltrate healthy brain tissue, and the presence of the blood–brain barrier, which limits drug penetration. Therefore, identifying novel molecular targets and therapeutic approaches is imperative to improving patient outcomes [3].

One promising avenue involves targeting the SP/NK‐1R signaling system, which has been implicated in tumor progression in various cancers, including glioblastoma [4]. The NK‐1R, a G protein‐coupled receptor (GPCR), is widely expressed in the human central and peripheral nervous systems, as well as in the digestive and respiratory tracts [5]. This receptor and its primary ligand, SP, play key roles in mediating pain perception, inflammatory responses, and stress regulation [6]. Notably, SP exhibits high affinity for NK‐1R, leading to its designation as the “SP receptor.” Importantly, the SP/NK‐1R interaction has been shown to drive glioblastoma cell survival, proliferation, migration, and angiogenesis while having minimal effects on healthy cells, making it an attractive therapeutic target. The NK‐1R is involved in the viability of U87‐MG when the silencing of the Tachykinin Receptor 1 (TAC1R), which encodes the NK‐1R, siRNA gene was carried out. That is, the expression of the NK‐1R is needed for the viability of U87‐MG cells [7].

Since its Food and Drug Administration (FDA) approval in 2003 as a selective NK‐1R antagonist for the prevention of chemotherapy‐induced nausea and vomiting, aprepitant has garnered attention for its significant anticancer potential. Notably, Muñoz and Rosso (2010) were the first to demonstrate its broad‐spectrum antitumor activity, showing that aprepitant induces dose‐dependent growth inhibition and apoptosis across multiple human cancer cell lines, including glioma, thereby emphasizing its antitumor effects mediated through NK‐1R blockade [8]. Studies indicate that aprepitant exposure reduces glioblastoma cell survival and proliferation, induces apoptosis in a dose‐dependent manner, and inhibits SP‐induced matrix metalloproteinase (MMP) production and migration—mechanisms associated with epithelial‐mesenchymal transition (EMT) and tumor progression [9, 10].

The current standard of care for glioblastoma, known as the Stupp protocol, combines surgical resection with concomitant radiotherapy and temozolomide, yet prognosis remains poor—highlighting the need for novel therapeutic strategies such as NK‐1R antagonism [11]. Given these insights, our study aims to further elucidate the molecular effects of aprepitant on glioblastoma metastasis using the U87 MG cell line. Specifically, we examined the expression and functional roles of NK1R and SP along with associated signaling pathways in this glioma model. These molecular targets were selected to deepen our understanding of aprepitant's mechanism of action at the cellular level and to explore its potential integration with the current Stupp protocol. The originality of this study lies in its focused expression‐based analysis of these oncogenic mediators and the investigation of aprepitant's tumor‐suppressive effects within a well‐characterized glioma model.

## 2 Materials and Methods

### 2.1 Chemicals

All chemicals and reagents were of analytical grade. RPMI‐1640, fetal bovine serum (FBS), phosphate‐buffered saline (PBS), dimethyl sulfoxide (DMSO), Trypsin–EDTA 10X, penicillin–streptomycin, L‐glutamine, SP, and Eagle's Minimum Essential Medium (EMEM) were purchased from Sigma Aldrich (St. Louis, MO, USA). Aprepitant was purchased from Santa Cruz Biotechnology (Santa Cruz Biotechnology, Delaware Ave, California, USA). BD Biosciences (San Jose, CA, USA) supplied the Matrigel. All other chemicals used were of analytical grade.

### 2.2 Cell Culture and Treatments

Human glioblastoma U87‐MG (American Type Culture Collection [ATCC], HTB‐14) cells were cultured in a Heracell CO_2_ incubator (Thermo Fisher Scientific, Pittsburgh, PA, USA) at 37°C (5% CO_2_, 95% humidity) with 10% FBS, 1% penicillin/streptomycin, and 1% L‐glutamine‐containing EMEM. Aprepitant was dissolved in DMSO and diluted to the required concentrations with fresh medium. Before the experimental procedures, cellular samples were subjected to staining with a trypan blue solution and subsequently assessed using a cell counting device (Cedex, Roche) [12]. The experiments were carried out utilizing viable cells in appropriate quantities. Aprepitant and SP were dissolved in DMSO. The control group was incubated in a 0.1% DMSO‐containing medium.

### 2.3 Determination of Cell Viability Using the WST‐1 Method

In our previous study, the IC_25_ (38.5 μM) and IC_50_ (77 μM) concentrations of aprepitant on U‐87 MG glioblastoma cells without SP were determined using MTT (3‐(4,5‐Dimethylthiazol‐2‐yl)‐2,5‐diphenyltetrazolium bromide) and real‐time cell analysis methods [9]. In this study, the effects of the same concentrations on U‐87 MG cell viability in the presence of SP were investigated using the WST‐1 method.

The U87‐MG cells were seeded into 96‐well plates at a density of 5 × 10^3^ cells/well. After 24 h of incubation, different concentrations of SP (5, 10, 50, 100, 500 nM) were applied, followed by the addition of the selected 500 nM SP concentration with aprepitant at IC_25_ and IC_50_ concentrations in designated wells. The plates were then incubated again for 24 h. 10 μL of WST‐1 reagent (Cat. no: 11644807001, Roche, Mannheim, Germany) was added to the cells, and the plates were incubated for an additional 3 h. At the end of the incubation period, absorbance values at 420 nm were measured for each concentration with 8 replicates (8 wells) using the Cytation 3 Cell Imaging Multi‐Mode Reader (Biotek). The results were analyzed using statistical analysis software [12].

### 2.4 Determination of the Anti‐Metastatic Effects of Aprepitant Using the Real‐Time Cell Analyzer System (RTCA)

The RTCA system (xCELLigence RTCA DP, Acea Biosciences) operates based on the principle of the Boyden chamber, utilizing impedance measurements for real‐time monitoring of cell motility. This system detects changes in electrical impedance as cells interact with the microelectrode surface, providing dynamic insights into cell adherence, proliferation, migration, and invasion. When cells adhere to the microelectrode‐coated wells, they increase electrical impedance, which correlates with their adhesion and spreading behavior. This label‐free, continuous monitoring approach enhances sensitivity and eliminates the need for staining, making it a valuable tool for assessing cell behavior under different conditions. The output, called the cell index (CI), reflects the specific cell properties being studied. The “Cell Invasion and Migration (CIM) plate” consists of a polyethylene terephthalate (PET) membrane with an 8 μm pore size and an ECM coating, facilitating the evaluation of cell invasion and migration [13].

To evaluate the anti‐metastatic effects of aprepitant, CIM plates with a two‐chamber system and a microporous membrane were used, either coated with Matrigel (for invasion) or without (for migration). After measuring the CI values of the background with the medium, 2 × 10^4^ U87‐MG cells per well were seeded into the top chamber of the CIM plates. Subsequently, aprepitant was applied to the wells at concentrations corresponding to previously determined IC_25_, IC_50_, and IC_75_ values of 38.5, 77, and 115.5 μM, respectively. Additionally, cells treated with 500 nM SP for 1 h before seeding were also subjected to aprepitant at IC_25_ and IC_50_ concentrations. The CIM plates were inserted into the device and programmed to take measurements every 10 min over a 24‐h period, initiating the experiment. Following 24 h from the application of concentrations, the device was stopped, and the analysis phase commenced. Invasion and migration curves were generated based on cell index values obtained with RTCA DP Software 1.2.1, and slope graphs were drawn using GraphPad Prism 7.0 software [14].

### 2.5 Determination of Protein Levels by ELISA

The levels of vascular endothelial growth factor (VEGF), interleukin‐6 (IL‐6), IL‐8, matrix metalloproteinase‐2 (MMP‐2), and MMP‐9 proteins involved in cancer pathogenesis were investigated using the ELISA method. U87‐MG cells were seeded into 6‐well plates at a density of 1 × 10^6^ cells/mL and incubated for 24 h. After incubation, some wells were subjected to a 1‐h pretreatment with 500 nM SP before being treated with aprepitant concentrations (IC_25_ and IC_50_) and further incubated for 24 h. Other wells were treated with aprepitant at the same concentrations but without prior treatment with SP. In the control groups, one set of cells was treated with medium containing 0.1% DMSO, while the other underwent a 1‐h pre‐treatment with 500 nM SP. After the incubation periods, supernatants collected from the samples were kept on ice, and protein measurements were conducted according to ELISA kit protocols. The kits used for the analysis were the Human VEGF‐A and Human IL‐8/NAP‐1 Platinum ELISA kits (BMS277/2 and BMS204/3, eBioscience, US), the IL‐6 ELISA kit (KAP1261, DIAsource, Belgium), and MMP‐2 and MMP‐9 ELISA kits (SEA100Hu and SEA553Hu, USCN Life Science, China). The colorimetric analysis of stained cells was performed using the Cytation 3 Cell Imaging Multi‐Mode Reader (BioTek), measuring absorbance at 450 nm [15].

### 2.6 Morphological Determination of Anti‐Migration Effects Using Oris Cell Migration Assay

Morphological migration analysis was performed with the Oris Cell Migration Assay kit (Platypus Technologies LLC., Madison, WI) following the manufacturer's instructions. This study utilized a cell migration assay kit, which included a special plate with stoppers for seeding cells. These stoppers ensured that the central area in all 96 wells remained empty during cell seeding. After seeding, the stoppers were removed from each well using a special tool, and this allowed for an equal area for cell migration in each well. In the 96‐well plate, U87‐MG cells were plated at a density of 5 × 10^4^ cells/mL, with each well containing 100 μL of medium. Following a 24‐h incubation period to facilitate cell adhesion to the plate, the medium was replaced, and a 1‐h pre‐treatment with 500 nM SP was performed before being treated with aprepitant concentrations (IC_25_ and IC_50_) at 0 and 24 h, and the stoppers were removed from the wells using the special tool. These wells were then stained with Hoechst 33,258 fluorescent dye, and images were captured at 0 and 24 h. Fluorescence intensity measurements in the range of 377–580 nm were also conducted and graphed using a Cytation 3 Multi‐Mode Reader (Biotek) [15].

### 2.7 Determination of Gene Expression Levels by RT‐PCR

The RT‐PCR method was used to study the mRNA expression levels of genes involved in cancer pathogenesis, including the NK‐1R, VEGF, nuclear factor kappa‐B (NF‐kB), tumor necrosis factor (TNF), IL‐1β, chemokine (C‐C motif) ligand 3 (CCL3), and chemokine (C‐X‐C motif) ligand 3 (CXCL3), which are particularly associated with inflammation, angiogenesis, and immune responses—key processes in cancer development and progression. U87‐MG cells were initially seeded in 6‐well plates at a density of 1 × 10^6^ cells in a medium containing 500 nM SP. After a 1‐h incubation, the medium was replaced with one containing IC_25_ and IC_50_ concentrations of aprepitant. The cells were then incubated for another 24 h. Total RNA was extracted from the U87‐MG cells using the MagNA Pure Compact RNA Isolation Kit (Roche, Lot: 13243700) and the MagNA Pure Compact Instrument LC 2.0 system (Roche Diagnostics, Mannheim, Germany). RNA quantity was measured spectrophotometrically at 260 nm and 280 nm using a NanoDrop 2000 (Thermo Scientific, USA). cDNA synthesis was performed following the Transcriptor High Fidelity cDNA Synthesis Kit (Cat. No. 05091284001, Roche) protocol. The synthesized cDNAs were amplified using a LightCycler 480 RT‐PCR instrument with the LightCycler 480 Probes Master Kit (Cat. No. 04707494001, Roche Applied Science, Germany). Both PCR mixtures and cDNAs were loaded into 96‐well plates and subjected to temperature settings suitable for the monochromatic hydrolysis probe method. The plate was then analyzed using the LightCycler 480 RT‐PCR instrument (Roche Applied Science, Germany). Glyceraldehyde‐3‐phosphate dehydrogenase (GAPDH) was used as the housekeeping gene, and gene expression levels were normalized to GAPDH using the SP4 version 1.5.0.39 software [16].

### 2.8 Microarray Experiments, Data Quality Control, and Analysis

For microarray analysis, total RNA was isolated using a total RNA isolation kit after incubating 1 × 10^6^ U87‐MG cells with 500 nM SP for 1 h, followed by replacing the medium with one containing the IC_50_ concentration (77 μM) of aprepitant and incubating for 24 h in 6‐well plates. The obtained RNA samples were analyzed using specialized microarray chips (Affymetrix Primeview Array) that contain known genes from the entire genome to profile the entire human genome and identify genes over‐ or under‐expressed in the experimental group compared to the control group (cells treated only with 500 nM SP).

The quality and concentration of total RNA were assessed using a NanoDrop 2000 (Thermo Scientific). For the application, 50–500 ng of total RNA was used. Single‐strand cDNA was synthesized, followed by double‐strand cDNA, then amplified RNA through in vitro transcription during a 16‐h incubation. The RNA sequences were labeled with biotin, and approximately 15 μg of labeled RNA was purified and fragmented. The fragmented material was prepared for hybridization to synthetic oligomers approximately 25 bp in length on the array. After incubation at 45°C for 16 h, arrays were washed and stained with a fluorescent dye (streptavidin phycoerythrin) to acquire signals from hybridized regions and remove non‐hybridized sequences. Scanning of washed and labeled arrays provided raw data from probe intensity values for each sample, processed and visualized using Affymetrix Gene Chip Command Console software. Microarray data analysis was performed using default settings in the Affymetrix Expression Console and Transcriptome Analysis Console [17].

### 2.9 Statistical Analysis

The data from each experiment was imported into GraphPad Prism 7.0 software. Replicates were averaged, and standard deviations were calculated. Graphics were generated using the same software. Statistical analysis was conducted using one‐way analysis of variance (ANOVA) followed by Tukey's post hoc test. Results represent the means of three independent experiments (*n* = 7 for ELISA assays, *n* = 3 for others) ± standard deviation (SD). Significance levels were indicated as follows: *p* > 0.05 non‐significant (n.s.); *p* < 0.05*; *p* < 0.01**; *p* < 0.001***; and *p* < 0.0001**** compared to the control group.

## 3 Results and Discussion

This study demonstrates, for the first time, that aprepitant exerts significant anti‐metastatic, anti‐proliferative, and anti‐angiogenic effects in U87‐MG glioblastoma cells. Our findings show that aprepitant not only reduces glioblastoma cell viability but also inhibits invasion, migration, VEGF production, inflammatory cytokine release, and the expression of critical metastasis‐associated genes. These results suggest that NK‐1R antagonism is a promising therapeutic strategy for glioma, potentially extendable to other tumors with high NK‐1R expression.

### 3.1 Evaluation of Cell Viability of Aprepitant Groups Induced With SP by WST‐1 Method

**FIGURE 1:**
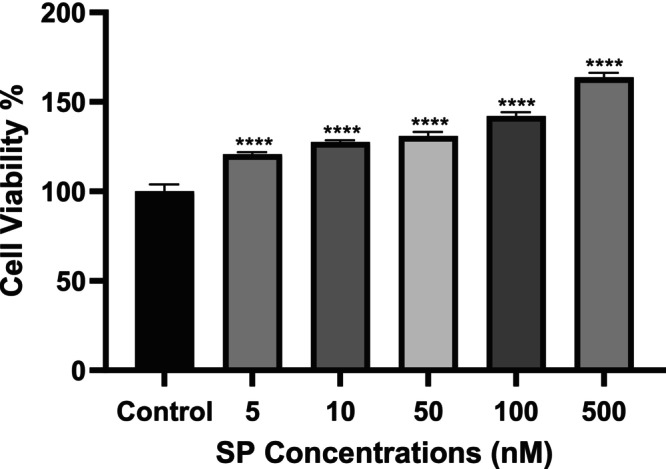
Evaluation of SP concentrations on U87‐MG cell viability. Cell viability was assessed by the WST‐1 method at 24 h and expressed as a percentage of the control. Statistical analysis was performed using one‐way ANOVA with Tukey's post hoc test, and the results are presented as mean values (*n* = 8) with significance levels indicated as *p* < 0.0001 (****). Data were analyzed relative to the control group (0.1% DMSO).

**FIGURE 2:**
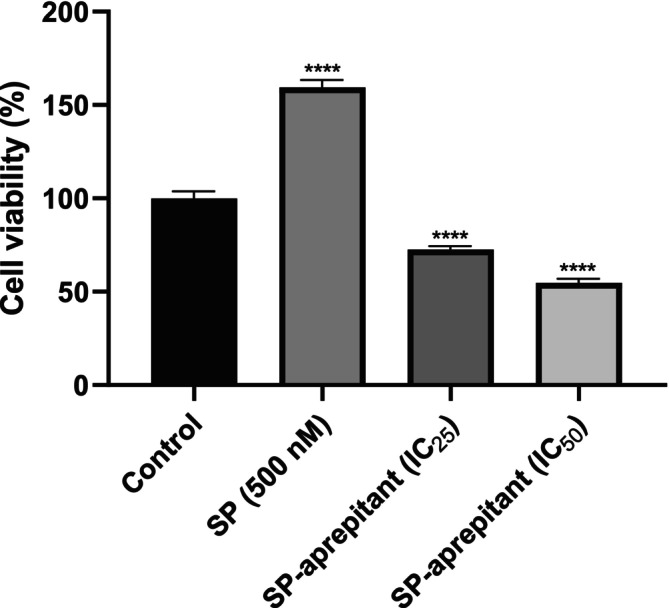
Evaluation of SP (500 nM) and aprepitant concentrations on U87‐MG cell viability. Cell viability was assessed by the WST‐1 method at 24 h and expressed as a percentage of the control. Statistical analysis was performed using one‐way ANOVA with Tukey's post hoc test. Results are presented as mean values (*n* = 8) with significance levels indicated as *p* < 0.0001 (****). Data were analyzed relative to the control group (0.1% DMSO).

A study was conducted to determine the proliferative effects of various concentrations of SP (5, 10, 50, 100, and 500 nM) on U87‐MG cells using the WST‐1 method. According to these results, the highest proliferation in U87‐MG cells compared to the control group was observed in the 500 nM SP group (Figure 1). Additionally, in our previous study [9], concentrations of aprepitant's IC_50_ (77 μM) and IC_25_ (38.5 μM) determined by MTT and real‐time cell analysis methods were combined with the 500 nM SP concentration, chosen due to its highest proliferation effect, to investigate the effects on U87‐MG cell viability. For this purpose, 500 nM SP was applied to the cells in the medium one hour before the application of aprepitant; then the medium was completely removed and replaced with a freshly prepared medium containing only the previously determined IC_25_ and IC_50_ concentrations of aprepitant. It was determined that the increased cell viability at a concentration of 500 nM SP in U87‐MG cells statistically decreased with the application of aprepitant concentrations (Figure 2).

These results expand on our previous work [9], where aprepitant significantly inhibited U87‐MG cell proliferation and induced apoptosis via caspase‐3 activation, supporting its role as a potential therapeutic agent targeting NK‐1R in glioblastoma.

SP is a peptide that exhibits selective affinity for NK‐1R and plays a crucial role in various physiological and pathophysiological processes, including neuroinflammation, emesis, mood regulation, and nociception [18]. In the present study, we confirmed that SP significantly enhances U87‐MG glioma cell proliferation, with the most potent effect observed at 500 nM (Figure 1). Similar findings have been reported in other glioma cell lines, such as GAMG, where SP also stimulates tumor cell proliferation [7].

In agreement with our results, previous studies have shown that SP promotes glioma proliferation in various cell lines, including GAMG and RG2, while NK‐1R antagonists such as L‐733,060 inhibit this effect both in vitro and in vivo [19, 20]. These findings collectively suggest that SP‐induced proliferation is a shared feature across multiple glioma models, emphasizing the therapeutic relevance of NK‐1R as a common target in glioma treatment.

Furthermore, in line with previous studies, NK‐1R antagonists, including aprepitant, exert antiproliferative and antitumor effects in various cancers, such as lung cancer [21] and osteosarcoma [22]. These results support the potential of NK‐1R blockade as a broad‐spectrum anticancer strategy.

### 3.2 Evaluation of Anti‐Metastatic Effects of U87‐MG Cells Using the Real‐Time Cell Analysis System (RTCA‐DP)

**FIGURE 3:**
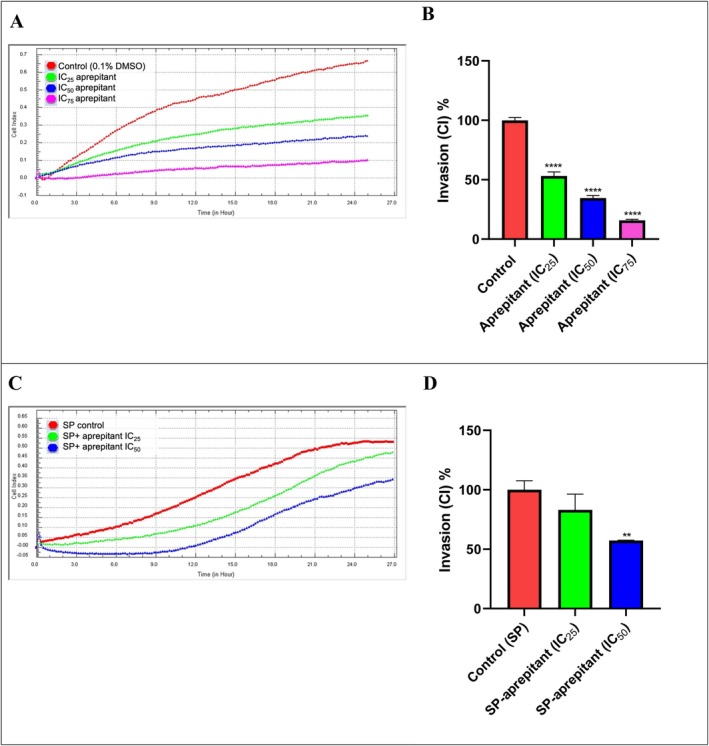
Invasion cell index (CI) curves from the RTCA‐DP system (A, C) and percentage slope graphs (B, D) were generated using the RTCA‐DP system to evaluate aprepitant concentrations (IC_25_: 38.5 μM, IC_50_: 77 μM, and IC_75_: 115.5 μM) alone and in combination with SP (500 nM) in U87‐MG cells. The percentage slope graphs (B and D) were plotted in GraphPad using the corresponding cell index curves (A, C). Data are presented as mean ± standard deviation (*n* = 6), with significance levels indicated as *p* < 0.01 (**), and *p* < 0.001 (***).

**FIGURE 4:**
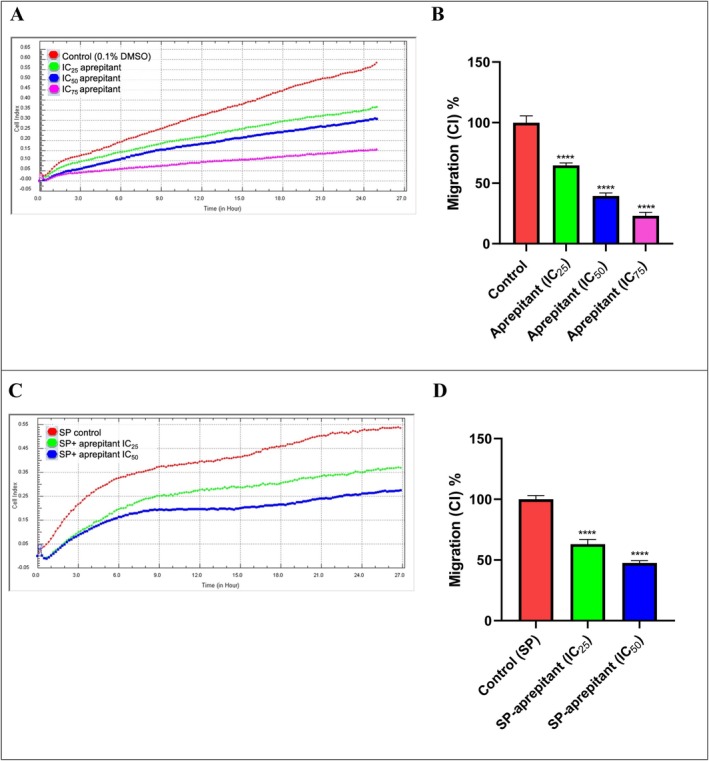
Migration cell index (CI) curves from the RTCA‐DP system (A, C) and the percentage slope graphs from GraphPad (B, D) were determined using the RTCA‐DP system for aprepitant concentrations (IC_25_: 38.5 μM, IC_50_: 77 μM, and IC_75_: 115.5 μM) alone and in combination with SP (500 nM) in U87‐MG cells. The percentage slope graphs (B, D) were plotted in GraphPad using the cell index curves (A, C). Data are presented as mean ± standard deviation (*n* = 6), with significance levels indicated as *p* < 0.0001 (****).

To evaluate the anti‐metastatic effects, invasion and migration methods were performed using the RTCA DP. In the invasion study, the effects of IC_25_ (38.50 μM), IC_50_ (77 μM), and IC_75_ (115.5 μM) concentrations of aprepitant alone and in combination with SP on cell invasion after a 24‐h incubation period were investigated based on proliferation curves in RTCA DP and slope graphs calculated with cell index values (Figure 3). Also, the effects of aprepitant concentrations, both alone and in combination with SP, on cell migration were investigated over a 24‐h incubation period using proliferation curves from the RTCA DP and slope graphs calculated with cell index values (Figure 4). As shown in Figure 3, an increase in aprepitant concentration resulted in a decrease in the invasion of U87‐MG cells, which possess invasive properties. Compared to the control, significant anti‐invasive effects were observed at aprepitant concentrations of IC_25_, IC_50_, and IC_75_ after a 24‐h incubation period (*p* < 0.001). In U87 MG cells treated with a combination of aprepitant and SP, a reduction in the invasion of metastatic U87‐MG cells was observed in correlation with increasing concentrations of aprepitant. Compared to the SP control group, the most significant anti‐invasive effect was observed at an aprepitant concentration of 77 μM (*p* < 0.01**).

As shown in Figure 4, an increase in the concentration of aprepitant alone resulted in a decrease in the migration of metastatic U87‐MG cells. Compared to the control, significant anti‐migratory effects were observed at aprepitant concentrations of IC_25_, IC_50_, and IC_75_ after a 24‐h incubation period (*p* < 0.001). Parallel to the invasion study, a decrease in the migration of U87‐MG cells stimulated with SP was observed with increasing concentrations of aprepitant. After a 24‐h incubation period, the most significant anti‐migratory effect compared to the SP control group was observed at an aprepitant concentration of 77 μM (*p* < 0.001***).

Our findings are consistent with previous research demonstrating that NK‐1R antagonists possess anti‐metastatic properties across multiple cancer types, not limited to glioma. For instance, aprepitant has been shown to inhibit proliferation, migration, and invasion in non–small‐cell lung cancer (NSCLC) models [21], as well as in MG‐63 osteosarcoma cells, where it suppressed tumor growth and metastatic behavior [22]. These data suggest that NK‐1R antagonism interferes with metastatic signaling pathways in a broad range of tumors.

Also, our findings are in agreement with previous studies highlighting the role of neurotransmitter‐induced tumor progression and metastasis, which can be pharmacologically inhibited by NK‐1R antagonists [23]. In particular, the involvement of tachykinins and NK‐1R in glioma growth and dissemination has been well established [24], further supporting the therapeutic rationale for targeting this pathway.

In the present study, we used SP pre‐treatment to mimic a tumor microenvironment with high SP levels, a protocol consistent with previous studies [4, 21, 22, 25]. By administering aprepitant after SP stimulation, we confirmed its capacity to interrupt SP‐mediated pro‐metastatic signaling, leading to reduced invasion and migration in U87‐MG glioma cells. Given the consistency of these findings across different tumor models, our results further support the therapeutic potential of NK‐1R antagonism, particularly with aprepitant, as a broad‐spectrum anticancer strategy.

### 3.3 Assessment of VEGF Levels Using the ELISA Method

**FIGURE 5:**
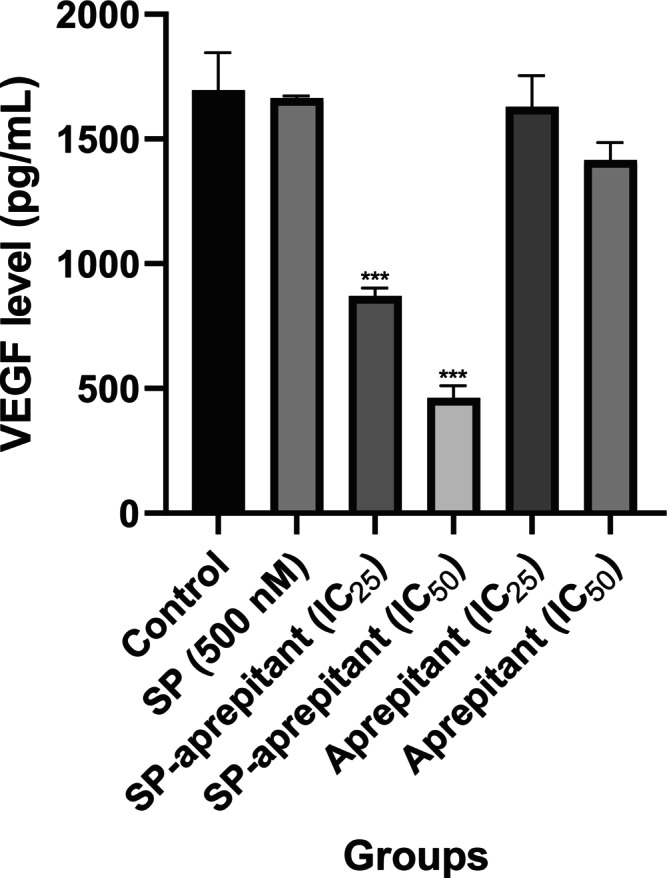
VEGF levels were determined by the ELISA method. The results are expressed as mean values (*n* = 7) in pg/mL, with significance levels indicated as *p* < 0.001 (***). Statistical analysis was performed relative to the control group (control: 0.1% DMSO, SP control: 500 nM SP, SP‐aprepitant IC_25_: SP (500 nM) + aprepitant IC_25_ (38.5 μM), SP‐aprepitant IC_50_: SP (500 nM) + aprepitant IC_50_ (77 μM)).

To investigate the relationship between aprepitant and angiogenesis, VEGF levels in the supernatants of U87‐MG cells incubated for 24 h with IC_25_ and IC_50_ concentrations of aprepitant were determined using the ELISA method. To quantify VEGF, a standard curve was first constructed based on the analysis of known VEGF standards in the ELISA. These standards, included in the kit and with known VEGF concentrations, were used to prepare the curve. Subsequently, from the VEGF standard graph, the VEGF levels corresponding to the absorbance of samples from control, SP, and aprepitant‐treated U87‐MG cell supernatants were calculated. The U87‐MG cells exhibited the highest levels of VEGF at 1664.667 ± 8.3 pg/mL and 1695.167 ± 151.3 in the 500 nM SP and control groups, respectively. There were notable decreases in VEGF levels in the groups that used SP in combination with IC_25_ and IC_50_ concentrations of aprepitant when compared to the control and SP control groups (*p* < 0.001***). In the groups where aprepitant concentrations were applied alone, there was a slight decrease in VEGF levels at the IC_50_ concentration of aprepitant compared to the control group. However, this decrease did not reach statistical significance (Figure 5).

VEGF is a well‐established regulator of tumor angiogenesis [26]. Overexpression of VEGF and its receptors (VEGFR) plays a critical role in promoting both angiogenesis and metastasis across various tumor types [27]. In alignment with our ELISA findings, previous studies have shown that NK‐1R antagonists, including aprepitant, effectively reduce VEGF and VEGFR expression in tumor models, exerting potent anti‐angiogenic effects [28]. This supports the hypothesis that aprepitant may counteract SP‐induced VEGF upregulation in glioblastoma.

To further investigate the relationship between the SP/NK‐1R pathway and angiogenesis, we assessed the effects of SP and aprepitant on VEGF levels in U87‐MG cells. Interestingly, aprepitant alone did not significantly reduce VEGF levels, which may be explained by basal NK‐1R activation in glioblastoma cells in the absence of exogenous SP. SP acts as a strong activator of NK‐1R signaling, leading to VEGF overexpression. Therefore, in the SP‐aprepitant group, the inhibition of VEGF was more pronounced due to the blockade of SP‐induced NK‐1R activation. In contrast, in the aprepitant‐only group, where endogenous SP levels are likely lower, the inhibition was less evident.

### 3.4 Evaluation of IL‐6 and IL‐8 Levels by ELISA Method

**FIGURE 6:**
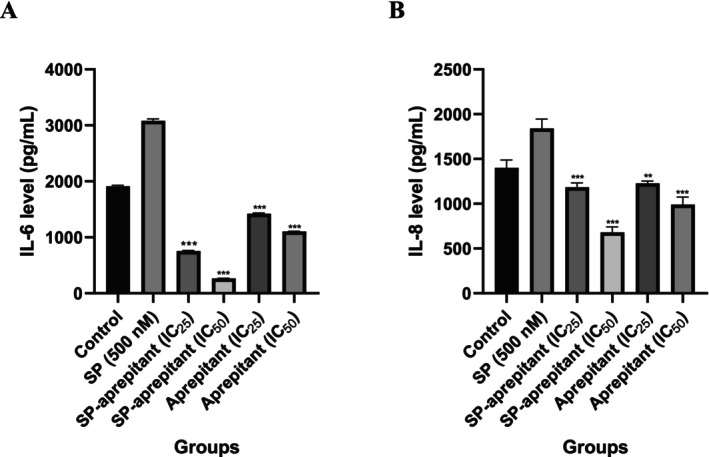
IL‐6 (A) and IL‐8 (B) levels were determined by the ELISA method. The results are expressed as mean values (*n* = 7), with significance levels indicated as *p* < 0.01 (**) and *p* < 0.001 (***). Statistical analysis was performed relative to the control group (control: 0.1% DMSO, SP control: 500 nM SP, SP‐aprepitant IC_25_: SP (500 nM) + aprepitant IC_25_ (38.5 μM), SP‐aprepitant IC_50_: SP (500 nM) + aprepitant IC_50_ (77 μM)).

In this study, the effects of aprepitant alone and in combination with SP on IL‐6 and IL‐8 levels in U87‐MG cells were determined using the ELISA method. A standard curve graph was plotted based on the absorbance analyses of the standards included in the kit to quantify IL‐6 and IL‐8. Subsequently, the levels of IL‐6 and IL‐8 in the U87‐MG cell supernatant samples treated with aprepitant and SP concentrations were determined from the standard graphs. The results are presented in Figure 6. According to the results, the highest IL‐6 and IL‐8 levels in U87‐MG cells, compared to the control group, were observed in the SP control group. Notably, the elevated IL‐6 and IL‐8 levels in the SP group were significantly reduced in the IC_25_ and IC_50_ aprepitant groups (*p* < 0.001***).

The functional role of IL‐6 and IL‐8 in glioma progression is well recognized. Both cytokines contribute to tumor growth, immune evasion, and angiogenesis by promoting a pro‐tumorigenic microenvironment. IL‐6 acts as a growth and survival factor for glioma cells [29, 30], while IL‐8 facilitates angiogenesis and enhances glioma cell migration [31]. These cytokines are not only markers of inflammation but also key players in glioma aggressiveness.

### 3.5 Evaluation of MMP‐2 and MMP‐9 Levels Using the ELISA Method

**FIGURE 7:**
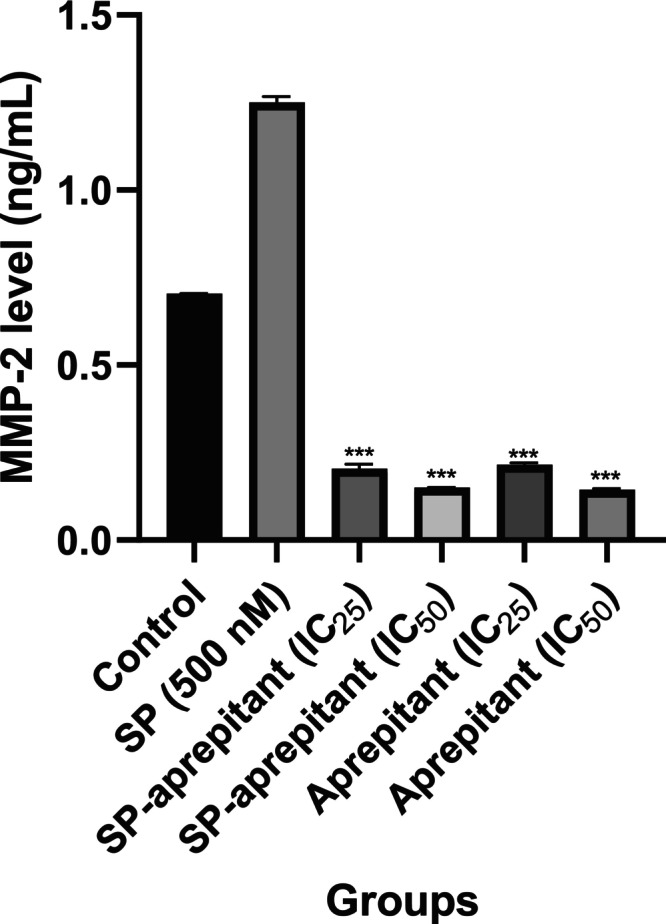
MMP‐2 levels determined by the ELISA method. The results are expressed as mean values (*n* = 7) in pg/mL, with significance levels indicated as *p* < 0.001 (***). Statistical analysis was performed relative to the control group (control: 0.1% DMSO, SP control: 500 nM SP, SP‐aprepitant IC_25_: SP (500 nM) + aprepitant IC_25_ (38.5 μM), SP‐aprepitant IC_50_: SP (500 nM) + aprepitant IC_50_ (77 μM)).

To determine the amounts of MMP‐2 and MMP‐9, the kit procedure was followed according to the standards provided within the kit, and standard curves were generated based on the absorbance and quantities of the MMP‐2 and MMP‐9 standards at 450 nm. Subsequently, based on the absorbance values obtained from the MMP‐2 standard graph, the amounts of MMP‐2 in the samples from each group were calculated and are presented in Figure 7.

According to the results, the highest MMP‐2 levels in U87‐MG cells, compared to the control group, were observed in the SP control group, with a value of 1.25 ± 0.017 ng/mL. In the groups treated with SP and IC_50_ aprepitant concentrations, the absorbance values measured for MMP‐2 were found to be lower than the absorbance of the 0.156 ng/mL standard included in the kit. Therefore, these values are reported as < 0.156 ng/mL in the table. The elevated MMP‐2 levels induced by SP were significantly reduced in all aprepitant groups compared to the control group (*p* < 0.001***). In U87‐MG cells, the absorbance values for MMP‐9 were measured to be lower than the absorbance associated with the lowest standard concentration. As a result, the MMP‐9 levels in all groups were calculated to be below 0.312 ng/mL. Therefore, the differences in MMP‐9 levels between the control and aprepitant groups could not be determined.

MMPs play a crucial role in the disintegration of basement membrane components and ECM. Research has indicated that higher levels of MMPs are linked to the advancement of cancer, higher invasion of tumor cells, and the spread of cancer to other parts of the body [32]. Furthermore, MMPs play a role in promoting tumor angiogenesis by breaking down capillary basement membranes and angiogenic cytokines like VEGF [27]. Previous studies have indicated that the heightened presence of MMP‐2 and MMP‐9 plays a crucial role in the development of various cancers and associated phenomena like invasion, metastasis, and angiogenesis [33].

MMP‐9 levels in all groups, including control and SP‐treated samples, remained below the assay's detection limit (0.312 ng/mL). This suggests that U87‐MG cells produce very low levels of MMP‐9 under basal conditions. Previous studies report that MMP‐2 is the predominant metalloproteinase in glioma cells such as U87‐MG, while MMP‐9 expression is generally absent or requires additional stimuli like hypoxia, phorbol esters, or stromal co‐culture. The absence of such stimuli in our model likely explains the low MMP‐9 levels observed [33].

### 3.6 Evaluation of Anti‐Migration Morphologically by Oris Cell Migration Assay

**FIGURE 8:**
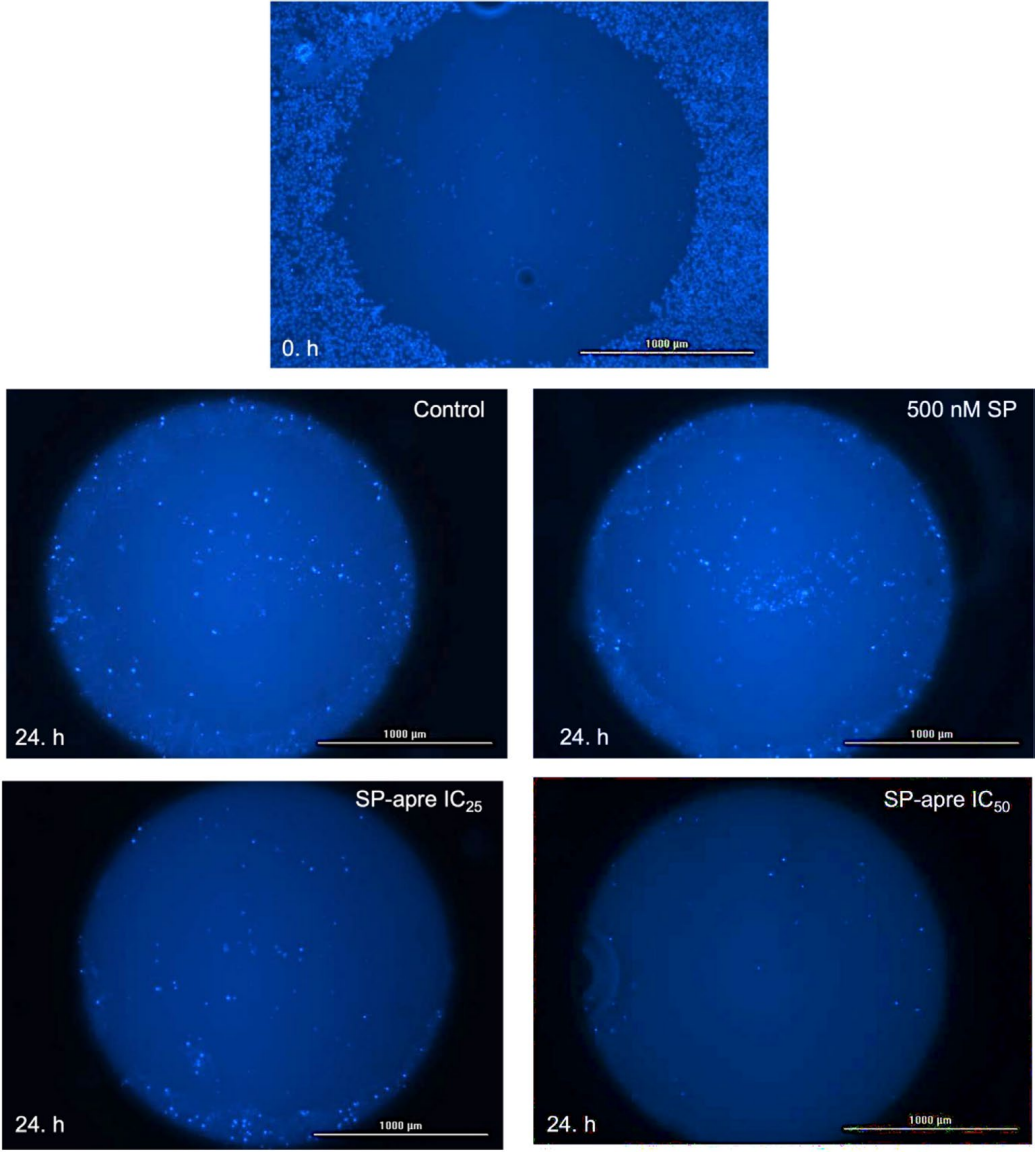
Morphological migration analysis results. The 24‐h impact of control, SP, SP‐aprepitant IC_25_, and SP‐aprepitant IC_50_ on U87‐MG cell migration is visually presented (objective: 10X). The image of the well at 0 h was taken from a randomly selected well after the removal of the stopper apparatus following the application of SP and aprepitant concentrations. Before capturing images of the wells at 24 h, a mask was applied to delineate the well dimensions as observed at 0 h.

**FIGURE 9:**
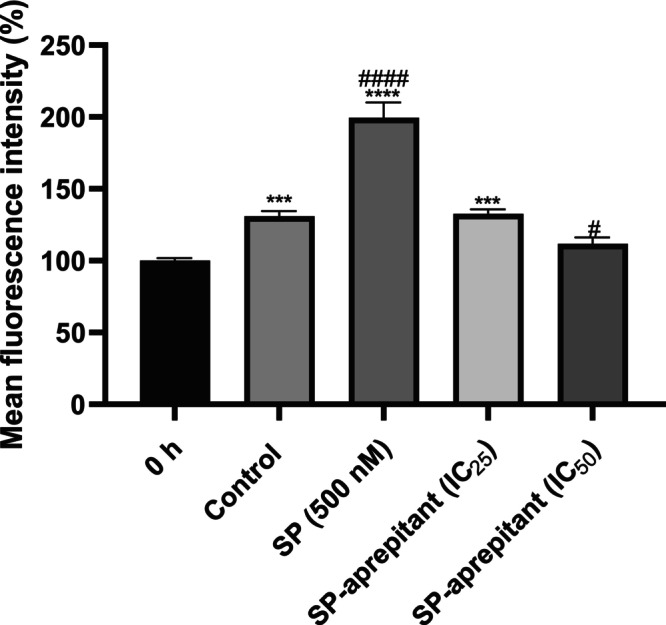
Morphologic analysis of migration. The column graph depicts cell migration across different concentration groups at 24 h, expressed relative to the mean fluorescence intensity measured across all groups at hour 0. Data are presented as means ± SD from three independent experiments (*n* = 3 per group). Statistical analysis was performed using one‐way ANOVA, followed by Tukey's post hoc multiple comparisons test (no difference: *p* > 0.05 ns; significant difference: *p* < 0.001 (***), and *p* < 0.0001 (****) according to the 0 h group; *p* < 0.05 (#), and *p* < 0.0001 (####) according to the control group at 24 h).

The migration experiment was supplemented with a morphological assessment (Figure 8). The column graph depicts cell migration across different concentration groups at 24 h, expressed relative to the mean fluorescence intensity measured across all groups at hour 0 (Figure 9). When examining the immunofluorescence intensity data representing U87‐MG cell migration at 24 h, the changes compared to hour 0 in the SP control, SP‐aprepitant IC_25_, and SP‐aprepitant IC_50_ groups were determined to be 130.84, 199.41, 132.67, and 111.78%, respectively. The increase in cell migration observed in the SP group was significantly inhibited in all experimental groups at 24 h, with the most pronounced effect in the SP‐aprepitant IC_50_ group, where cell migration levels decreased below those of the control group (#*p* < 0.05). Although the morphological experimental results are consistent with those obtained from real‐time cell migration analysis, the morphological analysis exhibits slightly lower sensitivity.

The Oris cell migration assay is a widely used technique to study cell migration, and it plays a crucial role in in vitro cancer research. This assay is particularly valuable for assessing the ability of cancer cells to move and spread to surrounding tissues, which are key aspects of metastasis [34].

### 3.7 Evaluation of mRNA Expression Levels of Genes Using the RT‐PCR Method

In this study, the expression levels of certain genes related to chemotaxis, angiogenesis, and signal transduction in U87‐MG cells were determined using the RT‐PCR method. The results were normalized to GAPDH expression levels and presented as averages of three experiments. The mRNA expression levels of the NK‐1R, VEGF, NF‐kB, TNF‐α, IL‐1β, CCL3, and CXCL3 genes in the groups were analyzed in comparison to the control group (Table 1). In U87‐MG cells, the expression of the NK‐1R gene increased 2.5‐fold in the SP control group compared to the control group. Following SP stimulation, downregulation of the NK‐1R gene was determined by PCR in groups treated with aprepitant at IC_25_ and IC_50_ concentrations, relative to the SP control group.

**TABLE 1:** Normalized analysis ratios of genes. This table presents the normalized analysis ratios of genes involved in cancer pathogenesis. Gene expression was analyzed in U87‐MG cells following treatment with SP and aprepitant at various concentrations (IC_25_, IC_50_). Data are normalized to the housekeeping gene GAPDH and presented as the ratio of gene expression relative to the control.

Genes normalized ratio ± standard deviation	Control	SP control	SP‐aprepitant (IC_25_)	SP‐aprepitant (IC_50_)
NK‐1R	1.000 ± 1.86E‐4	2.536 ± 2.83E‐4	1.338 ± 1.79E‐5	0.6305 ± 9.58E‐5
VEGF	1.000 ± 0.5085	1.157 ± 0.8160	0.9358 ± 0.4100	0.7973 ± 0.2931
NF‐kB	1.000 ± 7.36E‐2	8.852 ± 0.3202	5.823 ± 0.2277	1.051 ± 1.88E‐2
TNF‐α	1.000 ± 2.51E‐5	5.612 ± 7.70E‐5	3.873 ± 9.10E‐5	0.2797 ± 2.51E‐5
IL‐1β	1.000 ± 2.56E‐3	12.39 ± 4.45E‐2	5.625 ± 1.57E‐2	0.8020 ± 2.26E‐3
CCL3	1.000 ± 4.11E‐4	4.729 ± 1.54E‐3	3.383 ± 3.04E‐3	1.032 ± 2.59E‐4
CXCL3	1.000 ± 2.08E‐4	478.8 ± 0.1011	8.558 ± 4.16E‐3	0.7720 ± 1.66E‐4

Several genes modulated by SP/NK‐1R signaling play critical roles in glioblastoma progression. VEGF is a central driver of tumor angiogenesis, and its upregulation facilitates vascularization and tumor growth [35]. NF‐kB is constitutively active in glioma and acts as a key regulator of pro‐tumorigenic gene expression, promoting invasion, angiogenesis, and inflammation [36]. TNF‐α contributes to glioma cell proliferation and survival while maintaining a pro‐inflammatory tumor microenvironment [37]. Similarly, IL‐1β enhances glioma cell migration and invasion and induces VEGF secretion, further supporting angiogenesis [38]. IL‐6, through activation of the IL‐6/Janus Kinase (JAK)/Signal Transducer and Activator of Transcription 3 (STAT3) pathway, promotes glioma invasion, proliferation, and immune evasion [29]. CCL3 facilitates glioma cell migration and microglial recruitment, contributing to an immunosuppressive niche [39]. Finally, CXCL3 promotes glioma cell migration and angiogenesis via C‐X‐C Chemokine Receptor 2 (CXCR2) activation, enhancing the aggressive behavior of glioma cells [40]. These findings highlight the complex network of cytokines and chemokines driving glioma malignancy and underscore the potential of NK‐1R blockade to disrupt these pro‐tumorigenic pathways.

Regarding NK‐2R and NK‐3R, while SP can bind these receptors with lower affinity, current literature suggests that SP‐mediated receptor upregulation is largely selective for NK‐1R. There is limited evidence supporting significant SP‐induced NK‐2R or NK‐3R upregulation in glioma models. Studies have shown that NK‐2R and NK‐3R may be involved in other physiological responses, but the mitogenic, pro‐angiogenic, and anti‐apoptotic actions of SP in tumors are primarily mediated through NK‐1R [22, 41, 42]. Therefore, the selective upregulation of NK‐1R by SP, as observed in our study, is consistent with this receptor's dominant role in tumor biology.

Collectively, these findings support the notion that SP promotes glioma aggressiveness not only by triggering cell proliferation and migration but also by amplifying its own receptor pathway, reinforcing the potential of NK‐1R antagonists like aprepitant as therapeutic agents in glioblastoma.

### 3.8 Evaluation of Microarray Analysis

Our microarray analysis revealed significant gene expression changes in the SP‐aprepitant IC_50_ group compared to the control; particularly in genes related to chemotaxis, angiogenesis, and inflammation.

For microarray analysis, total RNA was isolated from samples of the U87‐MG control group and the SP‐aprepitant IC_50_ group (U87‐MG cells incubated for 24 h with the IC_50_ concentration of aprepitant following 1 h of SP stimulation). The obtained RNA samples were analyzed using specialized microarray chips (Affymetrix PrimeView Array) that contain known genes from the entire genome. The microarray analysis identified genes with at least 4‐fold upregulation and downregulation, and their functions are summarized (Table 2). The microarray analysis revealed upregulation of the RAS p21 protein activator 4 (RASA4) and tissue inhibitors of metalloproteases inhibitor 3 (TIMP3) genes and downregulation of the tripartite motif containing 5 (TRIM5), fibroblast growth factor 2 (FGF2), IL‐6, CCL20, CXCL2, IL‐11, IL‐8, CXCL5, CXCL3, CXCL1, and interleukin 1 receptor‐like 1 (IL1RL1) genes compared to the control group. These expression changes were predominantly observed in genes related to chemotaxis, angiogenesis, and signal transduction, aligning with the objectives and other findings of our study.

**TABLE 2:** Changes in genes determined in the SP‐aprepitant IC_50_ group compared to the control by microarray analysis. Data are shown as upregulation (+) or downregulation (−) of genes involved in cancer pathogenesis.

Genes	ANOVA *p*	Fold changes	Function
RASA4 (RAS p21 protein activator 4)	0,003052	(+) 5.36	Signal transduction
TIMP3 (TIMP metallopeptidase inhibitor 3)	0,000079	(+) 5.22	Neurodevelopment, MMP inhibitor
TRIM5 (tripartite motif containing 5)	0,004135	(−) 4.04	Immune response
FGF2 (fibroblast growth factor 2)	0,000197	(−) 4.05	MAPK kinase activity
IL6 (interleukin 6)	0,000065	(−) 4.22	Immune response, cell proliferation
CCL20 (chemokine (C‐C motif) ligand 20)	0,001212	(−) 4.57	Chemotaxis
CXCL2 chemokine (C‐X‐C motif) ligand 2	0,000131	(−) 4.65	Chemotaxis
IL11 (interleukin 11)	0,001612	(−) 5.16	Cell‐to‐cell signaling, immune response
IL8 (interleukin 8)	0,000007	(−) 8.47	Immune response, angiogenesis
CXCL5 (chemokine (C‐X‐C motif) ligand 5)	0,000478	(−) 8.68	Chemotaxis
CXCL3 (chemokine (C‐X‐C motif) ligand)	0,000118	(−) 12.12	Chemotaxis
CXCL1 (chemokine (C‐X‐C motif) ligand 1)	0,002127	(−) 15.19	Chemotaxis
IL1RL1 (interleukin 1 receptor‐like 1)	0,001174	(−) 17.16	Immune response

One of the key findings was the downregulation of TRIM5. Microarray analysis revealed a 5.22‐fold increase in the TIMP3 gene in the SP‐aprepitant IC_50_ group compared to the control in U87‐MG cells. TRIM5 is known to activate immune‐related pathways, including the NF‐kB signaling cascade, which can promote inflammation and potentially support tumor progression by creating a pro‐survival environment for tumor cells. The suppression of TRIM5 expression by aprepitant may therefore contribute to the observed decrease in NF‐kB expression and reduction in inflammatory signaling in our study [43]. This suggests that aprepitant not only inhibits tumor cell migration and invasion but may also reduce tumor‐promoting inflammation. Also, TIMPs regulate matrix‐degrading metalloproteases involved in various normal and pathological processes, including matrix turnover and cell migration. When overexpressed, TIMPs induce apoptosis and inhibit angiogenesis and cell migration. TIMP3 has been reported to reduce TNF‐α levels, thereby preventing inflammation [44]. Our findings support this information, as the increase in TIMP3 expression in U87‐MG cells incubated with 77 μM aprepitant for 24 h following a 1‐h SP stimulation parallels the decrease in TNF‐α expression observed in our RT‐PCR results.

In our study, compared to the control group, we observed at least a 4‐fold downregulation in the TRIM5, FGF2, IL6, CCL20, CXCL2, IL11, IL8, CXCL5, CXCL3, CXCL1, and IL1RL1 genes. Notably, the downregulation ratios for the IL8, CXCL5, CXCL3, CXCL1, and IL1RL1 genes were 8.47, 8.68, 12.12, 15.19, and 17.16‐fold, respectively. The direct association of these genes with chemotaxis and metastasis mechanisms further supports our previous findings. The anti‐metastatic effects of aprepitant on U87‐MG glioblastoma cells identified in our study are also consistent with our microarray results. The IL1RL1 gene encodes a protein that belongs to the interleukin 1 receptor family. The most extensively researched member of the family is the pro‐inflammatory protein IL‐1β, which transmits signals via the IL1R1 and stimulates the formation of angiogenesis in different disease states [45]. Glioblastoma is an aggressive type of cancer with no radical treatment options. The mortality rate is significantly high within a few years following diagnosis. In most glioblastomas, IL‐6 synthesis increases, which serves as a significant growth and survival factor for the cancer cells [46].

Additionally, our data demonstrated a marked downregulation of several chemokines, including CCL20, CXCL2, CXCL3, CXCL5, CXCL1, and IL8. These chemokines are key mediators of chemotaxis, immune cell recruitment, and angiogenesis. For instance, CXCL1, CXCL2, CXCL3, and CXCL5 interact with CXCR2, promoting glioma cell migration, angiogenesis, and tumor‐associated inflammation [47]. IL‐8 (CXCL8) is one of the most potent pro‐angiogenic and chemoattractant cytokines secreted by glioma cells, contributing to vascularization and immune cell infiltration [31]. Also, the connection of IL‐8 with glioma pathology arises from its ability to stimulate angiogenesis. IL‐8 is a potent chemotactic member of the chemokine family for immune cells. VEGF and IL‐8 are the main mediators released by glioma cells to enhance tumor vascularization. Stimulation of glioma cells with SP induces the release of IL‐8 [25]. CCL20 recruits regulatory T‐cells and contributes to immune evasion in glioma [48]. The significant downregulation of these chemokines by aprepitant may lead to a reduced recruitment of tumor‐promoting immune cells, decreased neovascularization, and suppressed tumor cell migration, supporting the anti‐metastatic and anti‐inflammatory effects observed in our study. These findings align with our RT‐PCR and ELISA results, reinforcing the conclusion that aprepitant's anti‐tumor action is multifaceted, targeting not only tumor proliferation but also the inflammatory and chemotactic signals that drive glioma progression.

Given the multifaceted antitumor effects of aprepitant demonstrated in our study, including antiproliferative, anti‐angiogenic, and anti‐metastatic activities, future research should focus on integrating NK‐1R antagonists into current glioblastoma treatment strategies. A potential approach would be to evaluate aprepitant as an adjuvant to the Stupp protocol, which currently consists of temozolomide and radiotherapy. Combining aprepitant with standard therapies may enhance treatment efficacy by targeting glioma invasiveness and therapy‐induced resistance pathways. Furthermore, clinical translation of aprepitant for oncology use would require well‐designed preclinical animal studies, followed by early‐phase clinical trials to confirm its antitumor efficacy and optimal dosing in glioblastoma patients. Since aprepitant is already FDA‐approved as an antiemetic with a favorable safety profile, drug repurposing represents a feasible and promising avenue for rapid clinical application.

## 4 Conclusions

In our study, significant anti‐metastatic (anti‐migration and anti‐invasion) effects of aprepitant on U87‐MG glioblastoma cells were identified. Our findings confirm that NK‐1R is a critical regulator of glioblastoma cell proliferation, migration, and invasion. Additionally, we demonstrated that aprepitant modulates the expression of multiple key genes and proteins involved in glioma progression, including VEGF, NF‐kB, TNF‐α, IL‐6, IL‐8, CCL3, CXCL3, and TRIM5, indicating its potential to suppress glioma‐associated angiogenesis, inflammation, and chemotaxis.

Given that SP promotes glioma aggressiveness through NK‐1R signaling and that NK‐1R antagonism with aprepitant effectively inhibits these pathways, our results support the potential of NK‐1R antagonists as novel therapeutic agents in glioblastoma management. These findings warrant further investigation in preclinical and clinical settings to explore the integration of NK‐1R blockade into glioma treatment strategies.

## Author Contributions

**Selin Engür‐Öztürk:** investigation (equal), writing – original draft (equal), software (lead), review and editing (equal). **Elif Kaya‐Tilki:** investigation (equal), writing – original draft (equal). **Miriş Dikmen:** supervision (lead), conceptualization (supporting), methodology (lead), review and editing (equal).

## Conflicts of Interest

The authors declare no conflicts of interest.

## Data Availability

The data that support the findings of this study are available from the corresponding author upon reasonable request.
